# Sleep-Related Breathing Problem Trajectories Across Early Childhood and Academic Achievement-Related Performance at Age Eight

**DOI:** 10.3389/fpsyg.2021.661156

**Published:** 2021-06-29

**Authors:** Rebecca Harding, Elizabeth Schaughency, Jillian J. Haszard, Amelia I. Gill, Rebekah Luo, Carmen Lobb, Patrick Dawes, Barbara Galland

**Affiliations:** ^1^Department of Women's and Children's Health, University of Otago, Dunedin, New Zealand; ^2^Department of Psychology, University of Otago, Dunedin, New Zealand; ^3^The Centre for Biostatistics, University of Otago, Dunedin, New Zealand; ^4^Department of Surgical Sciences, University of Otago, Dunedin, New Zealand

**Keywords:** academic achievement, obstructive sleep apnea, school performance, school grades, snoring

## Abstract

**Background:** Childhood sleep disordered breathing (SDB) has been linked to poorer academic performance; however, research has not investigated the extent improvement in SDB may alter outcomes across key academic skills. This study aimed to investigate if children's early SDB status could predict later academic outcomes, and if an improvement in SDB status across the early childhood years would coincide with better, later performance in key academic skills related to reading, numeracy, and listening comprehension.

**Methods:** Eighty five case children with an SDB symptom score >25 (maximum 77) were matched to 85 control children (score <12) at recruitment (age 3). SDB severity (symptom history and clinical assessment) was evaluated at ages 3, 4, 6, and 8 years and performance on individually-administered academic skills assessed at age 8 (91% retention from age 3). Case children were categorized into “improved” or “not-improved” groups based on SDB trajectories over the 5 years. Contributions of SDB status and trajectory group to academic performance were determined using regression analysis adjusted for demographic variables.

**Results:** History of SDB from age 3 predicted significantly poorer performance on some key academic skills (oral reading and listening skills) at age 8. Children whose SDB improved (45%) performed better in oral reading fluency than those whose SDB did not improve, but difficulties with specific tasks involving oral language (listening retell) remained when compared to controls.

**Conclusion:** Findings support links between early SDB and worse academic outcomes and suggest key academic areas of concern around oral language. Findings highlight the need for child mental health professionals to be aware of children's sleep problems, particularly SDB (past and present), when assessing potential barriers to children's achievement, to assist with appropriate and timely referrals for evaluation of children's sleep difficulties and collaborative evaluation of response to intervention for sleep difficulties.

## Introduction

Developmental psychologists are uniquely situated to contribute to our understanding of factors that contribute to children's educational experiences. To do so, requires awareness of pediatric health conditions that can create cognitive social-emotional and/or behavioral challenges for children, that in turn can potentially impact on how a child is functioning at school, including the acquisition of key learning skills for educational attainment. Good sleep health in children reflecting good quantity, quality, regularity and timing of sleep (Meltzer et al., 2021) is recognized as critical for children's optimal day-to-day functioning, developmental outcomes (Bernier et al., 2014), and developmental psychopathology (Gradisar et al., 2020), but many professionals across various specialties—including psychology—report limited education on adequately screening, diagnosing, and treating sleep problems (Drapeau, 2021). For example, childhood sleep disordered breathing (SDB), commonly reflected, and discussed, as chronic snoring (Perfect et al., 2014; Smith et al., 2017), has an overlapping presentation to ADHD in terms of symptoms of daytime hyperactivity (O'Brien et al., 2004), but is less well-recognized by mental health and educational professionals.

### Sleep Disordered Breathing and Academic Performance

Pediatric Sleep Disordered Breathing ranges from simple snoring to obstructive sleep apnea (OSA) (Mindell and Owens, 2015). The overall prevalence of SDB in school-aged children is ~10–12% (Marcus et al., 2012). The prevalence of OSA, in particular, is lower and varies by study, but those using gold-standard physiological assessment (polysomnography) for diagnosis report 1.2–5.7% (Marcus et al., 2012). Adenotonsillar hypertrophy (enlarged tonsils or adenoids) is the major cause of SDB in children, and consequently adenotonsillectomy is the most common treatment (Marcus et al., 2012). Craniofacial anomalies, obesity, and abnormal upper airway neuromotor tone are also implicated in the pathophysiology of SDB (Arens and Marcus, 2004). Previously, recognized sequelae were restricted to growth and cardiovascular complications, but accumulating evidence indicates cognitive and behavioral problems, and poorer school performance, are all associated with even mild SDB (Marcus et al., 2012). School performance, however, has received the least attention, but meta-analytic evidence derived mainly from cross-sectional studies supports a relationship between SDB and core academic subjects of language arts, math, science, and ratings of unsatisfactory progress or learning problems with standardized effect sizes ranging from −0.23 to −0.33 (Galland et al., 2015).

The majority of the studies in the field, however, have relied on parent or teacher report of global school performance, or school grades (Galland et al., 2015), rather than directly assessing important academic skills. In addition, because many of the studies in the SDB literature are cross-sectional, developmental implications over time cannot be understood (Bub et al., 2011). Establishing a temporal relationship between SDB and poorer academic performance is critical for understanding the best time, or window of opportunity, for intervention.

Two key studies published in 1998 and 2001, respectively, suggested that (a) early childhood SDB may predict poorer academic outcomes in later childhood, but that (b) academic difficulties were potentially reversible by treatment or natural resolution of SDB (Gozal, 1998; Gozal and Pope, 2001). However, studies examining this further have produced inconsistent findings, with several studies having methodological limitations. For example, before-after intervention studies have generally either demonstrated no statistically significant differences in academic achievement after treatment (Marcus et al., 2006; Esteller More et al., 2008; Biggs et al., 2014), or have not compared improvements to a control group or to expected academic growth (Giordani et al., 2012; Ikeda et al., 2012), limiting the conclusions that can be drawn. Longitudinal studies have produced mixed findings. Natural resolution of snoring a year after a first SDB assessment was related to improvements in behavioral functioning in third graders from Germany, but not to academic achievement (Urschitz et al., 2004). Similarly, a *retrospective* survey of Korean students found no evidence that the presence of past SDB (pre-school) was associated with school grades in elementary school (Kim et al., 2012), whereas a *prospective* New Zealand community survey found children who snored habitually at age 3, received lower parental ratings of literacy skills and overall academic performance at age 7, compared to those not identified to snore (Luo et al., 2018).

The American Academy of Pediatric guidelines for OSA treatment argue that “*the earlier a child is treated for OSAS, the higher the trajectory for academic and, therefore, economic success, but research is needed to support that implication”* (Marcus et al., 2012). However, the effect of adenotonsillectomy or other treatments on academic measures has not been sufficiently examined in children with SDB. The only published randomized controlled trial (RCT) of treatment of OSA did not report on academic performance specifically, although statistically significant improvements in functioning in the school setting were seen using teacher ratings (Marcus et al., 2013). RCTs in this field are challenging, giving importance to longitudinal studies to examine the temporal relationship between SDB and later academic outcomes.

### Methodological Considerations

Overnight polysomnography is considered the gold standard for SDB diagnosis. However, this is resource intensive, and therefore, for pragmatic reasons, researchers and general health professionals and child-serving professionals use other techniques (e.g., interview, questionnaires) to *screen* for possible SDB (Kemp, 2003; Luginbuehl and Bradley-Klug, 2008; Smith et al., 2017). Screening methods may also be combined to identify potential SDB (Goldstein et al., 2012).

Children's success with acquiring key academic skills, such as those involved in reading and math, in the early years (i.e., through age 8) are strong predictors of later academic success (National Research Council, 2015). Important skills involve those specific to each domain, such as reading text or computing mathematical operations (Silberglitt et al., 2016), but also general skills related to both, such as understanding and using language (National Research Council, 2015; Fuchs et al., 2021). Therefore, assessment of academic progress in the early years should include mastery of key domain-specific skills (Silberglitt et al., 2016) and related skills, such as comprehending and relaying information from spoken language (National Research Council, 2015; Lonigan and Burgess, 2017).

### The Current Study

The aim of this study was to investigate the relationships between SDB symptom trajectories screened across the pre-school to primary school years (ages 3, 4, 6, and 8 years) and academic performance outcomes at age 8 to answer the following questions: (a) does SDB in early childhood predict later performance on key academic skills related to reading, math, and listening comprehension assessed at age 8? and (b) does improvement in SDB symptoms offset potential associations with poorer performance?

## Methods

### Eligibility and Study Design

The study was approved by the NZ Lower South Regional Ethics committee (LRS/08/03/010). Recruitment involved a two-phase approach. Parents and caregivers of 3 year old children were first recruited from a survey of childhood snoring (Gill et al., 2012) that sought to identify the prevalence and health, sleep, and demographic factors associated with SDB in a community sample (small urban area in New Zealand's South Island; Dunedin). Parents of all children born between August 2004 and February 2006 who lived in the vicinity were invited to participate. There were no exclusion criteria other than living outside the greater Dunedin area. Of the 1,810 invitations sent, 839 participants (parents of children) completed and returned the questionnaire (response rate of 46.4%). Second, 823 respondents with complete data were asked for permission to be re-contacted for invitation to participate in the current longitudinal study on completion of the survey phase (80% response rate).

Exclusion criteria for entry at age 3 included extreme prematurity (<28 weeks), very low birth weight (<1.5kg), severe sensory or motor problems, cranio-facial abnormalities, major congenital abnormality, or significant chronic illness in the first 3 years of life. Eligibility criteria for inclusion at age 3 were: First, identification as potential cases (habitual snoring) and controls via parents' response to the survey question “How often does your child usually snore?” Potential cases parents responded “often” or always,” and potential controls “rarely” or “never.” Then, to maximize the difference between the Case and Control groups entering the study in terms of SDB severity risk, parents completed a SDB symptom questionnaire based on Goldstein et al. (2004) with items weighted according to their association with SDB; items 1–9 in Supplementary Table 1. Case children were eligible to enter on the basis of having an overall SDB score >25 (maximum 45) and control children potentially eligible on the basis of scores <12, pending matching on important demographic characteristics. Children whose scores fell between 12 and 24 were considered to be within an “uncertain” range for SDB symptoms and were excluded. Consequently 170 children and their families enrolled; firstly, identifying the 85 cases and then 85 controls matched one-to-one on gender, age (±2 months), body mass index (BMI) (z score ±1.5) and socio-economic status as measured by the New Zealand (NZ) Deprivation Index (decile ±3). Matching variables were prioritized in the order stated, and the proportion of pairs that met the matching criteria for each variable are as follows: gender (100%), neighborhood deprivation (97.7%), age (96.5%), BMI (91.8%).

The NZ Deprivation Index provides a deprivation score for geographical units defined by Statistics New Zealand, based on information from the national census on eight dimensions of deprivation. The deprivation scores are then categorized by deciles, so that areas have an index score that ranges from 1 to 10, where 1 represents areas with the lowest deprivation scores and 10 the highest deprivation scores (Salmond et al., 2007).

### Study Context

Children were then followed across four ages (3, 4, 6, and 8 years). Children in New Zealand enter school on their fifth birthday; therefore, age 8 follows 3 years of instruction in the education system, roughly equivalent to US 3rd Grade (NZ Year 4). Children attended various schools in the study community. Schools in New Zealand include state (a.k.a, public), state-integrated, or private schools, all of which receive some government curricular oversight and funding. The national New Zealand Curriculum (Ministry of Education, 2015) is taught in English-medium state (a.k.a., public) and state-integrated schools. State-integrated schools maintain their special character (usually a philosophical or religious belief) alongside the NZ Curriculum and receive the same per pupil funding as state schools.

### Participants

Parents reported their child's ethnicity, date of birth, street address [used to obtain deprivation index (Salmond et al., 2007)], and parental education levels. Children's height and weight were measured at each visit using a portable stadiometer and electronic scales. BMI (kg/m^2^) was calculated and percentiles determined using age- and sex-specific norms based on Center for Disease Control (CDC) growth charts (Kuczmarski et al., 2002). Table 1 reports descriptive statistics for participant characteristics across the 4 age periods. There was high participant retention with 154 of the original 170 participants at age 3 still in the study at age 8 (91%). Overall, children were predominantly New Zealand European/non-Māori (~88%), ~60% were male, mean BMI percentiles for age ranged from 61 to 74, ~79% lived in areas of medium to low deprivation and mothers' education levels were high (~72% with tertiary education or above). There were no statistically significant differences at age 3 in demographic characteristics between participants retained at age 8 (*n* = 154) compared to those who had dropped out by age 8 (*n* = 16).

**Table 1:** Descriptive statistics for demographic characteristics and BMI percentile at each time point.

**Variable**	**Age 3**	**Age 4**	**Age 6**	**Age 8**
	**(*n* = 170)**	**(*n* = 165)**	**(*n* = 163)**	**(*n* = 154)**
Age, mean (SD), y	3.7 (0.2)	4.7 (0.1)	6.3 (0.2)	8.0 (0.0)
Ethnicity, *n* (%)				
NZ European/Non-Māori	150 (88)	–	–	132 (87)
Māori	20 (12)	–	–	20 (13)
Sex, *n* (%)				
Male	100 (59)	97 (60)	97 (60)	91 (59)
Female	70 (41)	66 (40)	66 (40)	63 (41)
Deprivation index category^a^, *n* (%)				
Low (1–3)	66 (39)	65 (40)	65 (41)	60 (41)
Medium (4–7)	69 (41)	65 (40)	61 (38)	54 (37)
High (8–10)	35 (20)	33 (20)	33 (21)	33 (22)
Maternal education^b^, *n* (%)				
Secondary or below	50 (30)	43 (27)	44 (28)	40 (27)
Tertiary or above	119 (70)	116 (73)	115 (72)	108 (73)
Paternal education^b^, *n* (%)				
Secondary or below	87 (54)	83 (54)	88 (57)	67 (49)
Tertiary or above	75 (46)	70 (46)	66 (43)	69 (51)
BMI percentile, mean (SD)	70 (23)	74 (20)	71 (21)	61 (25)

*BMI, Body Mass Index*.

*–ethnicity not collected at ages 4 and 6*.

a *New Zealand Deprivation Index is a proxy measure of socioeconomic deprivation and ranges from 1 (least deprived) to 10 (most deprived)*.

b *Education level was measured on a scale from 1 (primary or below) to 5 (completed tertiary degree or diploma). A binary split was made for no tertiary and tertiary and above*.

### Measures

To address our research questions, measures included in this study were SDB symptoms and clinical evaluations assessed at each time point (ages 3, 4, 6, and 8) and academic performance assessed at age 8.

#### Sleep Disordered Breathing

At each of the four time points of the longitudinal study, children's SDB symptoms were measured using a clinical assessment score based on Goldstein et al. (2012). The assessment score included nine parent-report questions used at recruitment to this phase (see Supplementary Table 1; items 1–9) and six additional items (Supplementary Table 1; items 10–15) as part of a physical examination to create the total SDB assessment score ranging from 0 (asymptomatic) to 77 (indicating significant symptoms and features of SDB). The parent symptom questionnaire covers sleepiness, morning headaches, snoring, gasping, choking, sweating, enuresis, neck extension during sleep, and mouth breathing. The physical examination included the following objective measures: blood pressure percentiles; the presence or absence of mouth-breathing; ability to fog a mirror placed under the nose during normal breathing (latter two indicating presence or absence of nasal blockage commonly due to adenoid hypertrophy); the presence of adenoid facies (open mouthed, long face); tonsil size (graded-Brodsky scale); degree of hyponasality (the sound of speech with a blocked nose). In this sample, total parent ratings correlated with physical examination results at each time point (*r*'s = 0.25–0.35, *p*-values all < 0.01; Supplementary Table 1). Goldstein et al. (2012) found that combined parent-rated SDB questionnaire results and clinical examination of the child correctly identified 72% of children referred for overnight polysomnographic evaluation of SDB.

#### SDB Trajectories

To define SDB trajectories, we applied the same 15 SDB items making up our SDB score to data from a previous study where children's SDB was determined using gold standard polysomnography (Bradley et al., 2012) in a broader, but overlapping, age range (5–17 years). The data produced a moderately strong correlation between the SDB assessment score and polysomnographically-determined SDB (*r* = 0.52, *p* = 0.001) and correctly identified children with and without SDB with 75.0% sensitivity and 69.2% specificity. Using the receiver operator curves, children with a total severity score >20 were considered at high risk of SDB, whereas those <10 were considered at low risk, and between 10 and 20, at moderate risk. Thus, a difference of at least 10 points on the SDB severity scale could distinguish children at high risk of SDB compared to low. Over a 5-year period, for a child to go from high risk to low risk, this would equate to a drop of at least two points per year. This rate was used to categorize SDB children into those whose SDB had “improved” or “not improved” from ages 3 to 8. Intra-individual regression slopes were calculated for children with SDB at 3 years of age using SDB severity scores at age 3, 4, 6, and 8 years. A negative slope of at least −2 points per year or less was defined as having “improved,” whereas a slope greater than this was defined as “not improved.”

#### Academic Performance

At age 8, children completed reading, listening, and numeracy tasks collected over two field visits (~30–40 min each) by one author (CL) blinded to children's SDB scores.

##### Reading-Related Tasks

Reading tasks involved reading aloud three 3rd grade passages from the Dynamic Indicators of Basic Early Literacy Skills (DIBELS-Next)^1^ (Powell-Smith et al., 2010), scored for accuracy and fluency of oral reading (words read correctly per minute), followed by asking children to retell what they had read. Children's retell was audio-recorded for later transcribing and coding. Meta-analyses support oral reading fluency as an indicator of reading achievement (Reschly et al., 2009), and reading retell tasks have been found to correlate with performance on standardized tests of reading achievement in US samples (Roberts et al., 2005; Marcotte and Hintze, 2009). DIBELS oral reading and reading retell tasks have been shown to correlate moderately to strongly with standardized and school-used indicators of reading progress in New Zealand students in Years 3 and 4 (Schaughency et al., 2015, 2017). Reading retell was scored for length of retell (number of total words) and the extent the story was retold in the correct order (story sequence, see listening retell below). Number of total relevant words was derived from Systematic Analysis of Language Transcripts- New Zealand Version (Miller et al., 2012). For story sequence, two raters independently coded 25% of reading retell transcripts for each story to evaluate inter-rater reliability, with Cohen's Kappa (K) suggesting excellent agreement (*K* = 0.92–0.97). Median values from the three passages were used in analyses.

##### Listening-Related Tasks

Listening tasks were based on Westerveld and Heilmann (2012). Children first listened to an audio-recording telling the story depicted in the wordless picture book, *Frog Where Are You*? (Mayer, 1969). After the story, children were asked questions to assess their comprehension of the story (listening comprehension), then asked to retell the story. Retell coding was based on Maessen (2011) adaptation of the approach recommended by Reese et al. (2012). Transcripts of children's listening retell narratives were scored for how much of the story they retold (i.e., number of story propositions; story memory/narrative quantity) and the extent their retelling was told in the correct order (story sequence/narrative quality). Previous New Zealand research documents predictive relations between children's story memory and narrative quality in Year 2 (similar to 1st grade) and performance on a variety of reading tasks up to 2 years later (Schaughency et al., 2017). Inter-rater reliability evaluated from independent coding of 25% of the transcripts indicated substantial agreement between the coders for story comprehension (90% agreement), story memory (*K* = 0.84) and story sequence (*K* = 0.76).

Overall oral reading and listening retell scores were also calculated to reflect children's general performance in each area. Simple total scores would be inappropriate due to the dependent nature of scores within each domain [e.g., reading retell dependent on oral reading; story sequence dependent on story memory, see Reese et al. (2012)]. Therefore, each score was converted to quantiles to provide an index of relative performance within our sample. Children's mean quantile scores for the measures comprising each construct served as their overall score. Thus, overall oral reading was made up of oral reading fluency (words read correctly) and retell (number of total words and story sequence) variables. Quantiles for the three variables making up overall oral reading were correlated (*r*'*s* = 0.68–0.83, *p-*values all < 0.001). Quantiles for the listening retell variables of story memory and story sequence that made up overall listening retell correlated strongly, *r* = 0.73, *p* < 0.001.

##### Numeracy-Skills

Children's numeracy skills were assessed via four tasks from the National Education Monitoring Project (NEMP), a large-scale educational evaluation in NZ that included similarly-aged (Year 4) students (Ministry of Education, 2010). Tasks included in NEMP were considered to provide good representations of mathematical knowledge and skills (content validity) and designed for consistency in administration (Ministry of Education, 2010). We selected NEMP tasks: (a) to enhance validity related to response processes (American Educational Research Association et al., 2014), given potential relations between curriculum alignment and children's performance on assessment tasks (e.g., Good and Salvia, 1988); (b) to aid interpretation of our findings, given the availability of data on performance from a large sample (*n* = 1320) of NZ children (Ministry of Education, 2010); (c) to attend to social validity and credibility of findings for NZ educators (see Schaughency and Suggate, 2008; Schaughency et al., 2010). The four tasks included: addition, subtraction, long addition, and word problems. Word problems involved mathematical concepts of number, algebra, and measurement. The number of problems answered correctly were used in analyses. In addition, the percentage of correct responses was calculated for each task. Percentage correct on the four tasks correlated statistically (*r*'s 0.27–0.68; *p*-values = 0.002 to <0.001).

### Data Analyses

Initial descriptive statistics were calculated for all measures with central tendencies (dispersion) reported as the mean (standard deviation) or median (interquartile range; 25th to 75th percentile) if the data did not demonstrate an approximate normal distribution. Missing data were treated as missing at random. To investigate potential differential attrition, age 3 demographic characteristics of those who dropped out across the 5 years of study and those that were retained were compared using *t*-test for continuous variables and Fisher's exact test for categorical variables.

To determine differences in academic outcomes between groups at age 8, linear regression models were used with the academic score as the dependent variable, group as the independent variable, and adjusted for relevant demographic variables, i.e., maternal education, deprivation index, ethnicity, and gender (Biddulph et al., 2003). Both standardized and unstandardized academic outcomes were used. Mean differences, 95% confidence intervals and *p*-values were estimated from the models to aid in interpretation (Jaccard et al., 2006). As some data were skewed, the central tendency was better represented by medians and unstandardized differences between groups were also estimated using quantile regression for the difference in the medians. BMI was not included as a covariate due to its inclusion in the SDB severity score. Residuals of all models were plotted and assessed for homogeneity of variance and normality. Statistical significance is at the *p* < 0.05 level and there was no adjustment for multiple testing. All analyses were conducted using Stata version 14.2 (StataCorp, College Station, TX, USA).

### Power Analysis

The original matched case-control study was powered at 80% to detect a standardized difference in academic achievement of 0.3SD between cases (snorers) and controls (non-snorers) to a 5% significance level, assuming a within-pair SD of 0.6. This required that 72 pairs be recruited. To allow for 15% drop-out or incomplete data, 85 participants for each case and control group were recruited. This sample size then allowed for follow-up over the next 4 years: an estimated 25% drop-out would result in a sample size of 64 in each group with 80% power to detect (to 5% significance) an odds ratio of three for academic underachievement in snorers at baseline compared to non-snorers at baseline, given that 20% of children without SDB academically underachieve (Owens, 2009; Bonuck et al., 2011).

## Results

### SDB Severity Scores From Age 3 to Age 8

**Figure 1:**
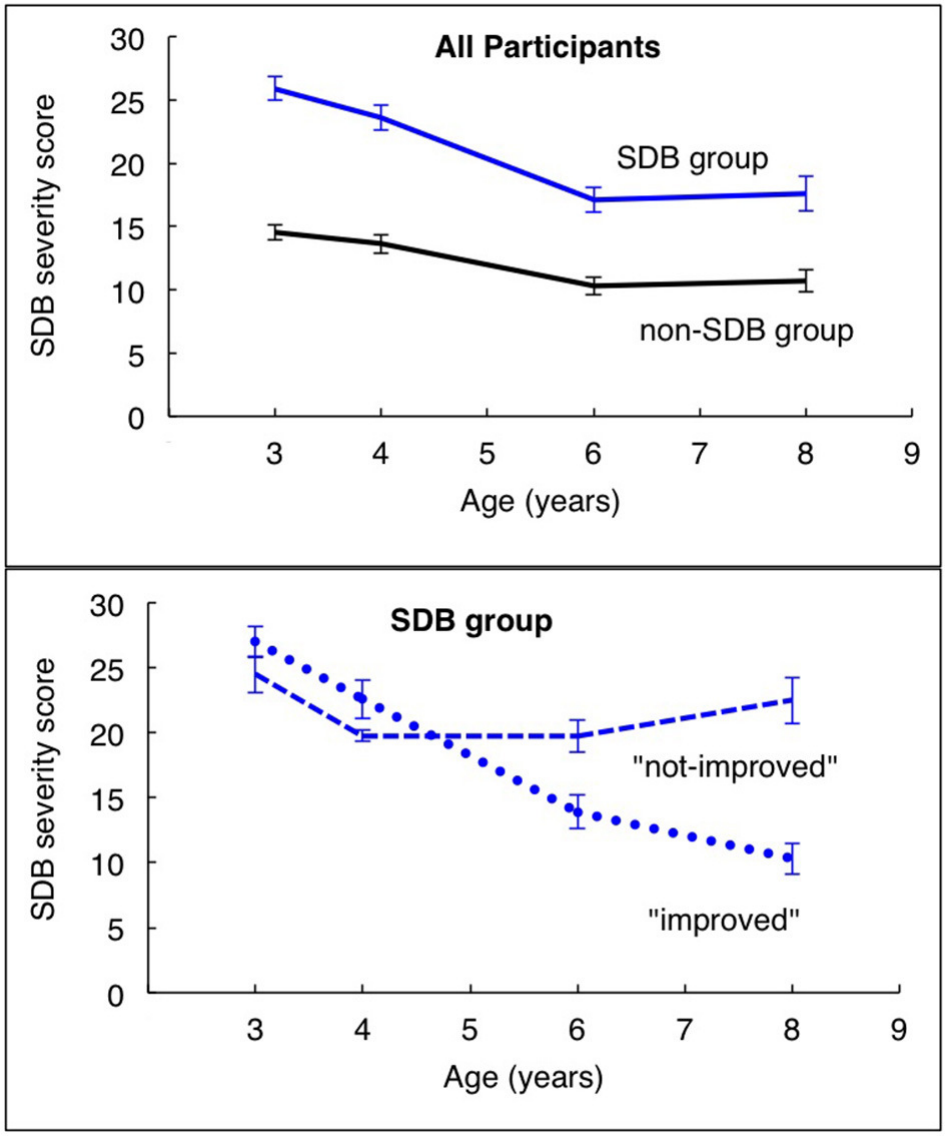
Mean ± sem SDB severity scores for each group evaluated across the four ages of assessment. Top graph represents SDB and non-SDB groups categorized by SDB severity scores at study entry (age 3). Bottom graph represents the SDB group further stratified by those whose scores had improved, or not, over the 5 year course.

Figure 1 illustrates the SDB severity scores across ages by group allocation into those with (n = 77) and without (*n* = 77) SDB symptoms at age 3 (top panel), and then into SDB sub-groups (i.e., “improvers” and “non-improvers”; bottom panel). Amongst children with SDB symptoms at age 3, 42 (55%) had *not shown* an improvement (“non-improvers”) in their SDB trajectory slopes across ages 3, 4, 6, and 8 years, whereas 35 (45%) had “improved.”

### SDB Status at Age 3 Predicting Academic Performance at Age 8

**Figure 2:**
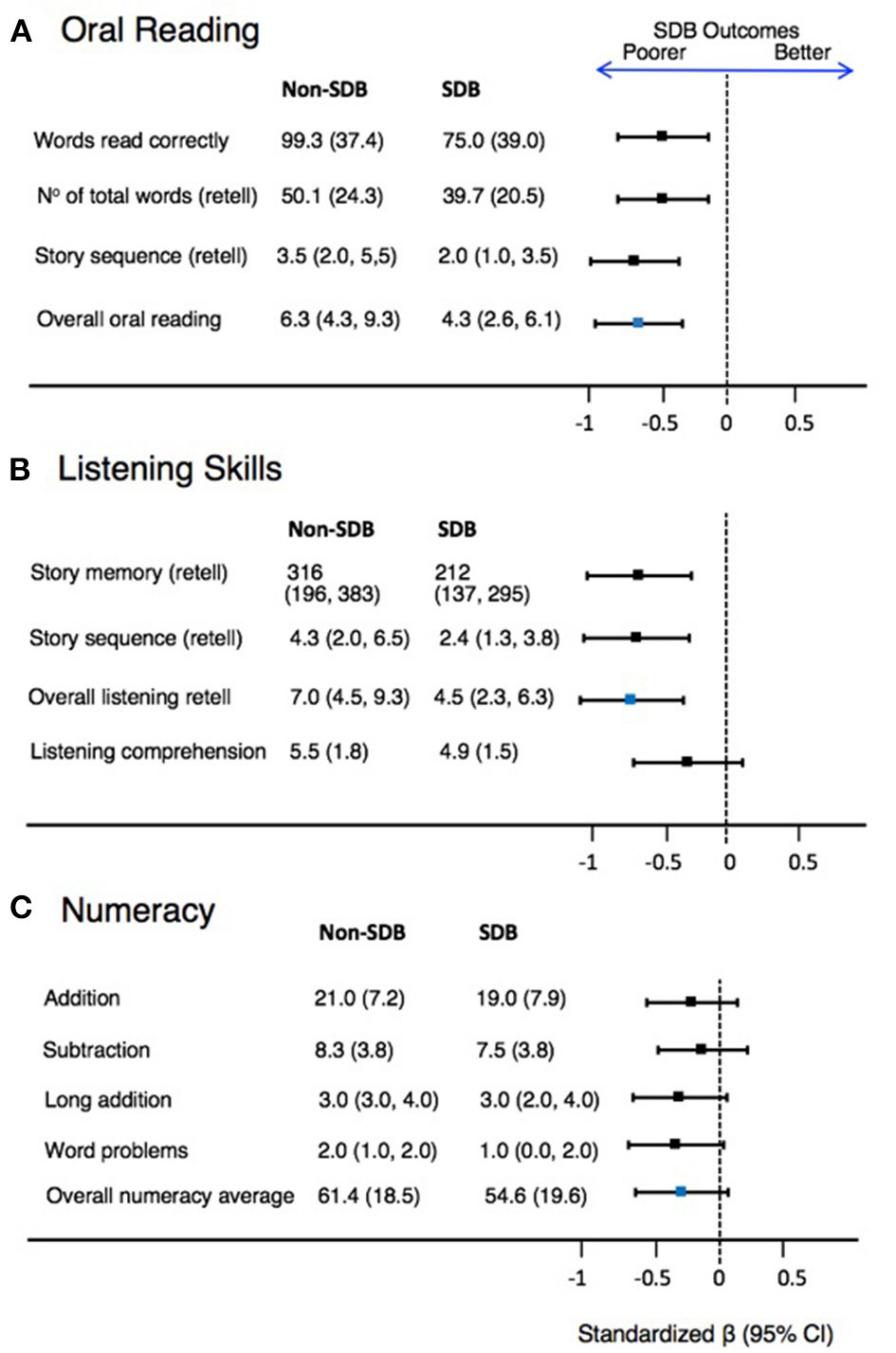
Descriptive statistics and standardized ß regression coefficients (bars represent 95% CI) for academic outcomes (panels **A** to **C**) at age 8 between SDB (cases) and non-SDB (controls) at age 3 (*n* = 143). Anything to the left of the dashed line (showing zero difference) indicates poorer academic outcomes at age 8 for those children who were identified to have SDB at age 3.

Descriptive statistics for academic skills scores and standardized regression coefficients (ßstd) representing the standardized mean difference are given in Figure 2 for children classified with SDB symptoms at age 3, compared to those who were not (non-SDB). Unstandardized regression coefficients (ß) representing the actual mean differences are given in Table 2. Those in the SDB group show evidence for poorer performance at age 8 for overall oral reading (Panel A): ßstd = −0.63 (95% CI −0.95, −0.32) and ß = −2.17 (95% CI −3.41, −0.93); *p* = 0.001) and overall listening retell skills (Panel B): ßstd = −0.67 (95% CI −1.04, −0.30) and ß = −2.0 (95% CI −3.55, −0.54); *p* = 0.012), with worse performance in all subtests making up these composites. There was little evidence for differences between groups for listening comprehension or overall average numeracy performance (although performance tended to be lower in the SDB group). While Figure 2 (panel C) illustrates the mean standardized differences (95% CI) in word problems between those with SDB vs. those without, the data for this outcome was not normally distributed and is better described by medians, with the difference in the medians assessed using quantile regression (Table 3). The difference in the medians (95% CI) for word problems between those with SDB and those without SDB was −1.0 (95% CI −1.58, −0.42); *p* = 0.001 (Table 2).

**Table 2:** Unstandardized regression coefficients (ß) representing mean/median differences in academic outcomes at age 8 between Sleep Disordered Breathing (SDB) and “non-SDB” groups.

	**Mean/median difference^a^ (95% CI)**	***P*-Value**
Oral reading fluency		
Words read correctly	−25.7 (−38.4, −13.0)	<0.001
N° of total words (retell)	−10.5 (−17.9, −3.14)	0.006
Story sequence (retell)	−1.50 (−2.51, −0.49)	0.004
Overall oral reading skills^τ^	−2.17 (−3.41, −0.93)	0.001
Listening		
Story memory (retell)	−110 (−187, −33.0)	0.006
Story sequence (retell)	−1.77 (−2.99, −0.54)	0.005
Overall listening retell skills^τ^	−2.0 (−3.55, −0.45)	0.012
Listening comprehension	−0.47 (−1.11, 0.17)	0.149
Numeracy		
Addition	−1.55 (−4.06, 0.97)	0.226
Subtraction	−0.49 (−1.73, 0.75)	0.434
Long addition	0.0 (−0.63, 0.63)	1.00
Word problems	−1.0 (−1.58, −0.42)	0.001
Overall numeracy^τ^	−5.39 (−11.9, 1.09)	0.102

a *Adjusted for maternal education, deprivation index, ethnicity, and gender*.

τ *Overall score for: Oral Reading (mean of the quantile scores of all three tasks); Listening (mean of the quantile scores for story memory and story sequence retell); Numeracy (mean of the percentage scores of the four math tasks)*.

*Lower/negative scores suggest worse academic performance in children*.

**Table 3:** Standardized regression coefficients (ßstd) for academic outcomes at age 8 between sleep disordered breathing (SDB) trajectory groups [“improved” (*n* = 35) and “not improved” (*n* = 42)] and non-SDB children (*n* = 77).

	**SDB “improved” vs. non-SDB**	***P*-value**	**SDB “not improved” vs. non-SDB**	***P*-value**	**SDB “improved” vs. “not improved”**	***P*-value**
Oral reading fluency						
Words read correctly	−0.35 (−0.73, 0.04)	0.075	−0.90 (−1.28, −0.51)	<0.001	0.61 (0.26, 0.96)	0.011
N^o^ of total words (retell)	−0.28 (−0.68, 0.12)	0.141	−0.62 (−1.02, −0.23)	0.002	0.38 (−0.06, 0.81)	0.087
Story sequence (retell)	−0.35 (−0.73, 0.03)	0.074	−0.94 (−1.31, −0.56)	0.001	0.60 (0.15, 1.05)	0.001
Overall oral reading skills^τ^	−0.34 (−0.72, 0.04)	0.078	−0.88 (−1.26, −0.50)	<0.001	0.56 (0.13, 0.99)	0.012
Listening						
Story memory (retell)	−0.57 (−1.03, −0.11)	0.015	−0.66 (−1.12, −0.21)	0.005	0.13 (−0.40, 0.65)	0.634
Story sequence (retell)	−0.64 (−1.11, −0.17)	0.008	−0.63 (−1.10, −0.17)	0.008	0.09 (−0.40, 0.58)	0.725
Overall listening retell skills^τ^	−0.62 (−1.09, −0.17)	0.008	−0.72 (−1.18, −0.26)	0.002	0.14 (−0.43, 0.71)	0.628
Listening comprehension	−0.27 (−0.76, 0.20)	0.257	−0.27 (−0.75, 0.21)	0.269	0.07 (−0.47, 0.62)	0.785
Numeracy						
Addition	−0.16 (−0.57, 0.25)	0.438	−0.21 (−0.62, 0.19)	0.302	0.10 (−0.41, 0.61)	0.694
Subtraction	0.00 (−0.40, 0.40)	>0.999	−0.23 (−0.64, 0.17)	0.255	0.27 (−0.23, 0.77)	0.282
Long addition	−0.21 (−0.62, 21)	0.324	−0.34 (−0.77, 0.08)	0.113	0.10 (−0.47, 0.67)	0.717
Word problems	−0.35 (−0.76, 0.07)	0.103	−0.36 (−0.79, 0.07)	0.098	0.02 (−0.48, 0.52)	0.927
Overall numeracy^τ^	−0.24 (−0.65, 0.17)	0.248	−0.30 (−0.73, 0.12)	0.730	0.07 (−0.45, 0.59)	0.797

a *Adjusted for maternal education, deprivation index, ethnicity, and gender*.

τ *Overall score for: Oral Reading (mean of the quantile scores of all three tasks); Listening (mean of the quantile scores for story memory and story sequence retell); Numeracy (mean of the percentage scores of the four math tasks). Lower/negative scores suggest worse academic performance in children*.

### SDB Symptom Trajectories Predicting Academic Performance at Age 8

Standardized regression coefficients (ßstd) for academic outcomes of children with SDB symptoms by SDB symptom trajectory (“improved” or “not-improved”) over the 5 years of study and those without SDB (non-SDB) are compared in Table 3. Descriptive statistics for academic scores are given in Table 4 and unstandardized regression coefficients in Table 5. Firstly comparing academic results for SDB “improvers” at age 8 (*n* = 35) with the non-SDB group (*n* = 77), performance for oral reading and listening comprehension was similar between groups. However, the SDB “improvers” displayed evidence of lower overall listening retell performance [ßstd = −0.62 and ß = −3.00 (95% CI −4.84, −1.16); *p* = 0.002] in both *story memory* [ßstd = −0.57 and ß = −108 (95% CI −201, −14.2); *p* = 0.024] and *story sequence* [ßstd = −0.64 and ß = −1.82 (95% CI −3.32, −0.26); *p* = 0.022].

**Table 4:** Difference in academic performance scores at age 8 by SDB trajectory group.

	**SDB “Improved” (*n* = 33)**	**SDB “Not-improved” (*n* = 37)**		
**Task**	**Mean (SD)/Median (25th, 75th percentile)**	**Mean (SD)/Median (25th, 75th percentile)**	**Mean difference (95% CI)^a^**	***P*-value**
Oral reading				
Words read correctly	86.0 (37.9)	64.8 (36.6)	−24.0 (−42.1, 6.0)	0.010
No of total words (retell)	44.2 (20.3)	35.1 (19.8)	−8.6 (−18.6, 1.3)	0.087
Story sequence (retell)	3.1 (1.9)	1.7 (1.5)	−1.4 (−2.2, −0.6)	0.001
Overall oral reading^τ^	5.3 (2.4)	3.8 (2.3)	−1.5 (−2.6, −0.3)	0.012
Listening^b^				
Story memory (retell)	216 (98)	214 (117)	−16 (−81, 50)	0.634
Story sequence (retell)	2.8 (2.1)	2.9 (2.3)	−0.2 (−1.6, 1.1)	0.725
Overall listening retell skills^τ^	4.6 (2.3)	4.5 (2.6)	−0.4 (−1.9, 1.2)	0.628
Listening comprehension	5.0 (0.9)	4.9 (1.9)	−0.1 (−1.0, 0.8)	0.785
Numeracy^c^				
Addition	20.0 (7.7)	19.3 (7.9)	−0.8 (−4.7, 3.1)	0.694
Subtraction	8.2 (3.8)	7.0 (3.7)	−1.0 (−2.9, 0.9)	0.282
Long addition^a^	3.0 (2.0, 4.0)	3.0 (2.0, 4.0)	0.0 (−0.9, 0.9)	>0.999
Word problems^a^	1.0 (0.0, 2.0)	1.0 (1.0, 2.0)	0.0 (−0.8, 0.8)	>0.999
Overall numeracy skills^τ^	56.1 (19.7)	54.1 (19.8)	−1.3 (−11.4, 8.8)	0.797

a *Difference in medians (95% CI) determined using quantile regression for those indicated; all adjusted for gender, household deprivation, Māori ethnicity, and maternal tertiary education*.

b *n = 25 in the “not improved” group and n = 30 in the “improved” group with listening scores*.

c *n = 32 in the “not improved” group and n = 33 in the “improved” group with numeracy scores*.

τ *Overall score for: Oral Reading (mean of the quantile scores of all three tasks); Listening (mean of the quantile scores for story memory and story sequence retell); Numeracy (mean percent correct scores of the four math tasks)*.

*Lower scores suggest worse academic performance in children*.

**Table 5:** Unstandardized regression coefficients (ß) representing mean differences in academic outcomes at age 8 between sleep disordered breathing (SDB) trajectory groups and non-SDB groups.

	**SDB “improved” vs. Non-SDB**		**SDB “not improved” vs. Non-SDB**		**SDB “improved” vs. “not improved”**	
	**ß (95% CI)^**a**^**	***P***	**ß (95% CI)^**a**^**	***P***	**ß (95% CI)^**a**^**	***P***
Oral reading fluency						
Words read correctly	−13.1 (−29.3, 1.44)	0.075	−35.8 (−51.1, −20.5)	<0.001	24.0 (5.98, 42.1)	0.010
N^o^ of total words (retell)	−6.41 (−15.5, 2.72)	0.167	−14.4 (−23.4, −5.28)	0.002	8.65 (−1.31, 18.6)	0.087
Story sequence (retell)	−0.50 (−1.73, 0.73)	0.422	−2.0 (−3.22, −0.78)	0.002	1.50 (0.50, 2.50)	0.004
Overall oral reading skills^τ^	−1.33 (−2.76, 0.09)	0.067	−3.0 (−4.42, −1.58)	<0.001	1.33 (−0.32, 2.99)	0.112
Listening						
Story memory (retell)	−108 (−201, −14.2)	0.024	−110 (−203, −16.8)	0.021	8.0 (−82.6, 98.6)	0.860
Story sequence (retell)	−1.82 (−3.32, −0.26)	0.022	−2.21 (−3.76, −0.66)	0.006	−0.11 (−1.71, 1.49)	0.890
Overall listening retell skills^τ^	−3.00 (−4.84, −1.16)	0.002	−2.50 (−4.33, −0.67)	0.008	−0.50 (−2.73, 1.73)	0.655
Listening comprehension	−0.46 (−1.25, 0.34)	0.257	−0.44 (−1.24, 0.35)	0.269	0.12 (−0.77, 1.02)	0.785
Numeracy						
Addition	−1.82 (−4.32, 1.88)	0.438	−1.63 (−4.75, 1.48)	0.302	0.77 (−3.12, 4.66)	0.694
Subtraction	−0.00 (−1.52, 1.52)	1.00	−0.39 (−2.42, 0.65)	0.255	1.02 (−0.86, 2.90)	0.282
Long addition	0.0 (−0.62, 0.62)	1.00	0.0 (−0.64, 0.64)	1.00	0.0 (−0.72, 0.72)	1.00
Word problems	−1.0 (−1.70, −0.30)	0.005	−1.0 (−1.72, −0.28)	0.007	0.0 (−0.77, 0.77)	1.00
Overall numeracy^τ^	−4.65 (−12.6, 3.28)	0.248	−5.87 (−14.0, 2.31)	0.158	1.30 (−8.79, 11.4)	0.797

a *Adjusted for maternal education, deprivation index, ethnicity, and gender*.

τ *Overall score for: Oral Reading (mean of the quantile scores of all three tasks); Listening (mean of the quantile scores for story memory and story sequence retell); Numeracy (mean of the percentage scores of the four math tasks)*.

*Lower/negative scores suggest worse academic performance in children*.

Secondly, for “non-improvers” (*n* = 42), oral reading skill performance was substantially lower than that of their non-SDB counterparts (*n* = 77) for overall oral reading [ßstd = −0.88, and ß = −3.0 (95% CI −4.42, −1.58); *p* < 0.001] and likewise for overall listening retell scores [ßstd = −0.72 and ß = −2.50 (95% CI −4.33, −0.67); *p* = 0.008], with significantly worse performance on most subtests making up these composites.

Third, comparisons between “improved” vs. “not-improved” SDB symptom trajectories indicated differences oral reading subtests of *words read correctly* per minute [ßstd = 0.61 and ß = −24.0 (95% CI 5.98, 42.1); *p* = 0.01] and *story sequence* [ßstd = 0.60 and ß = −1.50 (95% CI 0.50, 2.50); *p* = 0.004] with “improvers” demonstrating better performance at age 8 than “non-improvers.” The proportion of variance explained by the models when comparing the SDB group with the non-SDB group ranged from 5.7% (numeracy long addition) to 17.0% (listening comprehension); and when comparing the “improved” to the “not improved” group ranged from 4.0% (overall listening retell skills) to 27.9% (oral reading fluency story sequence retell).

## Discussion

Several findings emerged from this study. Children's SDB status at age 3 predicted poorer age 8 oral reading and retell (reading or listening) performance compared to their counterparts without SDB. Some numeracy and listening comprehension results were also lower, but results were not as consistent, suggesting SDB did not hinder performance on those tasks. However, results were dependent on whether children's SDB had, or had not, improved across childhood and the specific outcome measure. In this study, just over half of those with SDB at age 3 had improved SDB severity scores by age 8, whereas just under half had not. Thus, at age 8, improvement in SDB found children to be achieving similarly in basic oral reading and numeracy skills (computational tasks, excluding word problems) to those not displaying SDB symptomology at age 3, but listening retell performance was lower despite improved SDB (due to treatment or natural resolution). For those whose SDB status *did not improve* across childhood, performance on oral reading, listening retell and math word problems tasks were below that of peers with no SDB history at three, whereas basic numeracy skills (computational tasks excluding word problems) and accuracy of responses to listening comprehension questions were on par with those who had never had SDB.

These findings are consistent with, and extend, the results of the prospective NZ survey study that found habitual snoring in children at 3 years to be a unique, and significant predictor of poorer parent-ratings of their child's literacy skills at age 7 (Luo et al., 2018). The current study includes assessments of recommended literacy (oral reading and listening comprehension; Lonigan and Burgess, 2017) and measures of numeracy skills, enhancing interpretation of results. For example, oral reading fluency is a well-established curriculum-based measure of reading for this age group (Reschly et al., 2009). Mean reading fluency scores for children without a history of SDB in our study (*M* = 99; *SD* = 37) were consistent with those obtained in two different samples of similarly-aged children in our community, whereas mean fluency scores for children with a history of SDB (*M* = 75, *SD* = 39) were more similar to children in Year 2 in both studies (Schaughency et al., 2015, 2017). Likewise, children without a history of SDB (*Mdn* = 2; 25th percentile 1.0, 75th 2.0) performed similarly or better than 65% of a nationwide sample of Year 4 students on the word problem task, whereas children with a history of SDB (*Mdn* = 1; 25th percentile 0.0, 75th percentile 2.0), performed similarly or better than 35% of the NEMP Year 4 sample (Ministry of Education, 2010). The results are also consistent with previous research that identified school-age children with SDB to be more at risk for poorer performance in several academic domains, including unsatisfactory progress/learning problems (Galland et al., 2015; Harding et al., 2020).

In this study, children whose SDB severity scores improved from age 3-to-8 demonstrated higher oral reading fluency (words read correctly per minute) and reading retell (story sequence) skills at age 8, in comparison to children whose SDB symptom severity scores did not improve. Increased cognitive and academic performance have sometimes been reported following improved SDB symptom severity through tonsillectomy (Montgomery-Downs et al., 2005; Honaker et al., 2009; Giordani et al., 2012). The process by which SDB improvement may contribute to later reading acquisition is yet to be determined. Prior to school entry and formal reading instruction, the home literacy environment and early childhood education contribute to developing language and early literacy skills (Zauche et al., 2016). However, when children in our sample were 4 years of age, relations between children's early literacy skills and the early childhood literacy environment were moderated by children's parent-reported habitual snoring status at age 3. There was a dose-response relation between environmental exposure and children's skill development observed for children who *did not* have a history of snoring at age three, which was not evident in children with a past history of habitual snoring at age 3 (Luo et al., 2011). This could imply that SDB symptoms (i.e., habitual snoring) may interfere with the degree children benefit from their early childhood literacy learning experiences.

Importantly, the findings from this paper add the possibility that when SDB improves—thus potentially removing associated barriers to learning—developmentally important basic reading skills underlying fluent reading (e.g., decoding, word reading) may be attained. To directly examine this hypothesis longitudinally, future work should model children's developing skills alongside SDB symptoms. Such work presents methodological challenges, given need for changing assessment targets, from developmental precursor skills predictive of reading to reading following exposure to reading instruction (Bandalos and Raczynski, 2015). School-based practitioners could further contribute by monitoring children's skill development in the context of intervention for SDB (Maessen et al., 2021), consistent with the use of curriculum-based approaches to evaluate children's response to pharmacological intervention for ADHD (Stoner et al., 2002).

Children whose SDB symptoms improved across childhood (ages 3-to-8) did not reach academic competencies similar to peers without SDB symptoms at age 3 on all tasks, performing less well on listening retell quantity (*story memory)*, quality (*story sequence)*, and *numeracy word problem* tasks. These findings could suggest SDB presenting in early childhood may impact cognitive-linguistic skills involved in performance on these academic tasks, regardless of whether the children's SDB had been treated or resolved naturally over the intervening 5 years. Oral language skills are involved in comprehending and retelling a story (Schaughency et al., 2017) and in understanding and performing mathematics word problems (Peng et al., 2020; Fuchs et al., 2021).

To solve word problems, theory suggests children need to: (a) construct a coherent description of essential details from the problem, (b) supplement information provided with inferences based on background knowledge, including mathematical relations, and (c) coordinate this information to guide mathematical problem-solving (Fuchs et al., 2021). Thus, in addition to mathematical knowledge, solving word problems includes competencies similar to those involved in reading comprehension (Silverman et al., 2020). Solving word problems is conceptualized as a complex task involving oral language—and specifically narrative–skills and higher level cognitive processes related to executive-functioning and memory (Fuchs et al., 2021). It may be the higher level cognitive-linguistic skills involved in narrative and word problem tasks continued to be subtlety affected (see also Honaker et al., 2009; Giordani et al., 2012). In contrast, basic reading, math computation, and answering listening comprehension questions were intact at the time of outcome assessments, following 3 years of exposure to instruction.

Obtained links between SDB and academic performance, across childhood, may be partly dependent on when SDB and cognitive and academic skills are assessed. Oral narrative skills used in retelling stories continue to develop between 6-and-9 years of age (Peterson, 1983), and with further growth of these and other neurocognitive competencies, if assessed later, academic differences as a function of SDB history may no longer be detected. Given the importance of higher level cognitive-linguistic skills for meeting the increasingly sophisticated demands in reading comprehension (Castles et al., 2018), mathematical problem-solving (Peng et al., 2020), and learning across the subject areas (Foorman and Wanzek, 2016), observed educational impacts could also potentially widen over time.

Exact mechanisms linking SDB to poorer academic performance are not completely elucidated and likely complex. Potential biological pathways are posited to involve the intermittent hypoxia and sleep fragmentation associated with breathing pauses during sleep (characteristic of SDB), adversely affecting the prefrontal cortex and cognitive/executive control (Beebe and Gozal, 2002). Sleep fragmentation, in turn, can adversely affect sleep quantity, quality, and lead to daytime sleepiness. Each of these variables has been associated poorer academic performance (Dewald et al., 2010) and functioning (Liu et al., 2016; Smith et al., 2017).

Plausible pathways to poorer academic performance involve negative impact on cognitive functioning important for learning and school success, with cognitive correlates of SDB including verbal and non-verbal reasoning, and memory and executive functioning (Krajewski and Schneider, 2009; Prior et al., 2011; Blair et al., 2015), all of which can contribute to children's performance on narrative (Reese et al., 2012) and word problems tasks (Fuchs et al., 2021). The influence of a preschool-history of SDB on parent-reported academic performance in early elementary school has been suggested to be mediated through children's functional memory skills (Luo et al., 2018). Furthermore, SDB severity, as measured as in this study, contributed indirectly to a composite measure of academic performance assessed concurrently in young school-age children through performance and ratings on measures of executive functioning, verbal comprehension/communication and non-verbal reasoning (Luo et al., 2019). Despite these documented links between SDB symptoms and cognitive performance, possible pathways to poorer learning outcomes also include associations between SDB and learning-related behavioral functioning (Perfect et al., 2014).

### Strengths and Limitations

Our study had several strengths, including the high retention rate with only 16 participants (9%) withdrawing from the longitudinal study between age 3 and age 8, repeated measurement of SDB symptoms to describe the SDB trajectories of children over time to help elucidate symptom profiles, and the assessment of important academic skills at age 8. Individually-administered assessments of academic skills assessments used here provide more sensitive and specific information than broad indices of achievement (Katz and Slomka, 2000) and cover key areas of reading, math and listening comprehension to give a deeper understanding of performance in these specific skills. Although these assessments may not reflect day-to-day classroom performance when distractions are present, they complement other methods such as traditional achievement measures (e.g., reading comprehension) and real-world data (e.g., teacher ratings) in describing children's academic progress (Kettler and Albers, 2013).

Our study also had limitations. A limitation was the lack of polysomnography for determining SDB. We used parental report of symptoms combined with a physical examination of features associated with SDB to build the clinical assessment score. Identification of SDB based on clinical symptoms is a common approach used by many research studies directly exploring the relationship between SDB and cognitive or neuropsychological deficits (Marcus et al., 2012), and even in clinical practice, most children have adenotonsillectomies performed without having overnight polysomnography or any other physiological measure of SDB (Friedman et al., 2013). Parent reported severity of symptoms of SDB in young children have been shown to be a better predictor of behavioral and cognitive outcomes than polysomnography-derived indices (Perfect et al., 2014). Importantly, research is emerging that supports use of questionnaires for assessing SDB in the context of treatment planning and evaluation (Chan et al., 2019). Understanding predictive relations between SDB risk as assessed by screening methods and children's developmental outcomes adds to the evidence base for these assessment approaches. The items used to build the clinical assessment score reflect widely accepted and empirically-supported features and symptoms of SDB and the full scoring system itself has been used to determine SDB accuracy derived from polysomnography in our previous work (Bradley et al., 2012). A further limitation is the uncertain accuracy of SDB trajectory groups. These were categorized based on data from a sample of children and adolescents (5–17 year-olds) overlapping in age with participants in the current study. However, the mean SDB severity scores of “improved” and “non-improved” groups at age 8, were similar to the scores of 5–17 year olds at low and high risk of SDB, respectively. The gold-standard criteria for defining SDB is the same across the pediatric age ranges encompassed by both studies (Kaditis et al., 2016), and contributors to the pathophysiology of SDB in children occur independent of age (Dayyat et al., 2009). We also need to consider that, although our sample was drawn from a non-clinical community sample of children, initial purposive recruitment of participants at age 3 specifically targeted children with and without symptoms of SDB; therefore, study findings cannot be extrapolated to a general community sample. In addition, the predominantly NZ European/Non-Māori (87–88%) ethnic composition of the study sample limits the generalisability of findings to ethnic minority groups who are over-represented in the SDB statistics (Boss et al., 2011), including Māori (Gill et al., 2012). The scope of the study was also limited by the lack of information on potential influences on SDB and learning that were not assessed, such as interrupted learning because of changes in school, family circumstances or other health-related issues, as well as sample size and loss of a small number of participants across the 5 years of study.

### Conclusions

Overall, the data document that children with a history of SDB from early childhood performed worse than their non-SDB counterparts at age 8 on several academic measures including oral reading, listening skills and word problems. Results highlight the importance of understanding later academic consequences of early childhood SDB trajectories. Findings contribute to further understanding of the potential implications of persistent SDB on academic outcomes and suggest successful resolution of SDB may remove some barriers to learning and potentially benefit academic outcomes. Although our study provides a temporal framework for links between early childhood SDB and academic outcomes at age 8, the study cannot help determine the optimal time to treat SDB in children to mitigate academic sequelae. The latter will always be challenging because of the changing dynamics of SDB across childhood (Anuntaseree et al., 2005; Luo et al., 2015).

Child mental health professionals should be aware that sleep difficulties, such as those associated with SDB, may impact academic progress. Possible signs of SDB include mouth breathing, sleepiness, attentional difficulties, dysregulated behavior, and learning challenges. With raised awareness of SDB, child mental health professionals are in a prime position to facilitate appropriate screening and referral for medical consultation, partner with educational and medical professionals to evaluate response to intervention, and collaborate to address remaining learning needs, ultimately contributing to improved learning outcomes for children (Luginbuehl and Bradley-Klug, 2008). Findings that children whose SBD improved continued to show some academic challenges serve to highlight potential areas where children might benefit from instructional interventions, including oral language (Peng et al., 2020), narrative (Nicolopoulou and Trapp, 2018), and math word problems (Fuchs et al., 2021). Thus, children with ongoing SDB may need additional learning and behavioral supports as well as appropriate intervention for sleep difficulties.

## Data Availability Statement

The raw data supporting the conclusions of this article will be made available by the authors, without undue reservation.

## Ethics Statement

The studies involving human participants were reviewed and approved by New Zealand Lower South Regional Ethics committee. Written informed consent to participate in this study was provided by the participants' legal guardian/next of kin.

## Author Contributions

RH: data curation, statistical analysis, and writing–original draft. ES and BG: conceptualization, methodology, writing–review and editing, funding acquisition, and supervision. JH: overseeing statistical analysis and interpretation, supervision, and writing–review and editing. PD: conceptualization, methodology, and review and editing. AG and RL: conceptualization, methodology, data collection, and writing–review. CL: data collection and writing–review. All authors contributed to the article and approved the submitted version.

## Conflict of Interest

The authors declare that the research was conducted in the absence of any commercial or financial relationships that could be construed as a potential conflict of interest.

## Footnotes

^1^Dynamic Measurement Group. DIBELS Next. Dynamic Measurement Group.
