# Supplementary material for: Sleep-Related Breathing Problem Trajectories Across Early Childhood and Academic Achievement-Related Performance at Age Eight
Source: Front Psychol. 2021 Jun 29;12:661156. doi: 10.3389/fpsyg.2021.661156 (PMC8276242; doi:10.3389/fpsyg.2021.661156)
Supplement: Supplementary file 1 [file Table_1.docx]

**Supplementary Table 1:** Abbreviated sleep disordered breathing assessment scale items and scoring

| Items | Maximum possible score^a^ |  |  |  |  |
| --- | --- | --- | --- | --- | --- |
| Parent rated daytime and night-time SDB symptoms and correlates |  | Every night | 4 to 6 nights/wk | 1 to 3 nights /wk | Never |
| 1. How often does your child get sleepy during the day? | 6 | 6 | 4 | 2 | 0 |
| 1. How often does your child complain of morning headaches? | 3 | 3 | 2 | 1 | 0 |
| 1. How often does your child snore while he/she is asleep? | 6 | 6 | 4 | 2 | 0 |
| 1. How often does your child gasp for breath during sleep? | 6 | 6 | 4 | 2 | 0 |
| 1. How often does your child make choking noises while asleep? | 3 | 3 | 2 | 1 | 0 |
| 1. How often does your child sweat profusely during the night? | 3 | 3 | 2 | 1 | 0 |
| 1. How often does your child wet the bed? | 6 | 6 | 4 | 2 | 0 |
| 1. How often does your child sleep with his/her neck extended (stretched out)? | 6 | 6 | 4 | 2 | 0 |
| 1. How often does your child breathe through his/her mouth? | 6 | 6 | 4 | 2 | 0 |
| Subtotal | 45 |  |  |  |  |
| Physical examination |  |  |  |  |  |
| 1. Blood pressure (percentile) | 6 | ≥ 95^th^, 6 | ≥ 90^th^ and < 95^th^, 4 | < 90^th^, 0 |  |
| 1. The absence or presence of mouth breathing | 4 | Present, 4 | Absent, 0 |  |  |
| 1. The absence or presence of hyponasal voice | 4 | Present, 4 | Absent, 0 |  |  |
| 1. The degree of adenoid facies | 6 | Severe, 6 | Mild, 4 | Absent, 0 |  |
| 1. The ability to fog mirror placed under the nose during normal breathing | 6 | None, 6 | Poor, 4 | Fair, 2 | Well, 0 |
| 1. Tonsil size (graded according to the Brodsky scale) | 6 | 4+, 6 | 3+, 4 | 2+, 2 | 0-1+, 0 |
| Subtotal | 32 |  |  |  |  |
| Total | 77 |  |  |  |  |

^a^Maximum possible scores are based on Goldstein et al. (2012) where symptoms more specific to SDB are weighted more heavily than less suggestive symptoms. Correlations between parent rating and physical examination subtotals at each age were as follows: age 3: *r* = .29; age 4: *r* = .35; age 6: *r* = .25; age 8: *r* = .35, *p*’s < .01.
